# Secondary and Tertiary Transmission of Vaccinia Virus After Sexual Contact with a Smallpox Vaccinee — San Diego, California, 2012

**Published:** 2013-03-01

**Authors:** Hai Shao, Eric C. McDonald, Michele M. Ginsberg, Lisa M. Yee, Jay R. Montgomery, Frances Allan-Martinez, Mary G. Reynolds, Danielle M. Tack, Whitni Davidson, Nishi Patel, Rachael Joseph

**Affiliations:** Scripps Mercy Hospital, San Diego; Public Health Svcs, County of San Diego Health and Human Svcs Agency; Vaccine Healthcare Centers Network, US Department of Defense; Div of High-Consequence Pathogens and Pathology, National Center for Emerging and Zoonotic Infectious Diseases; EIS Officer, CDC

On June 24, 2012, CDC notified Public Health Services, County of San Diego Health and Human Services Agency, of a suspected case of vaccinia virus infection transmitted by sexual contact. The case had been reported to CDC by an infectious disease specialist who had requested vaccinia immune globulin intravenous (VIGIV) (Cangene Corporation, Berwyn, Pennsylvania) for a patient with lesions suspicious for vaccinia. The patient reported two recent sexual contacts: one with a partner who recently had been vaccinated against smallpox and a later encounter with an unvaccinated partner. Infections resulting from secondary transmission of vaccinia virus from the smallpox vaccinee to the patient and subsequent tertiary transmission of the virus from the patient to the unvaccinated partner were confirmed by the County of San Diego Public Health Laboratory. The smallpox vaccine had been administered under the U.S. Department of Defense smallpox vaccination program. The vaccinee did not experience vaccine-associated complications; however, the secondary and tertiary patients were hospitalized and treated with VIGIV. No further transmission was known to have occurred. This report describes the epidemiology and clinical course of the secondary and tertiary cases and efforts to prevent further transmission to contacts.

## Secondary Vaccinia Case

On June 24, a man went to a private hospital in San Diego County with a painful perianal rash of 3 days’ duration and more recent onset of a lesion on the upper lip. The patient reported having had sexual intercourse on June 15 with a man who had recently been vaccinated against smallpox. The patient recalled feeling moisture on an uncovered area of his partner’s left upper arm and was concerned that his rash might have been caused by this exposure.

In addition to the rash, the patient reported experiencing fever, malaise, nausea, and vomiting before seeking medical attention. He also reported a history of psoriasis and a possible history of eczema. Atopic dermatitis (i.e., eczema) can be a risk factor for adverse reactions to vaccinia infection (1,2). While performing the physical examination, the infectious disease specialist noted seven 5-mm umbilicated lesions in the perianal area and a similar lesion on the upper lip. The County of San Diego Public Health Laboratory detected nonvariola *Orthopoxvirus* by polymerase chain reaction (PCR) on swab specimens from the lesions.

The patient was hospitalized for continued care and observation. Human immunodeficiency virus and other sexually transmitted infections were ruled out during his hospitalization. VIGIV was requested from CDC and administered intravenously on June 25 because of concerns about the location and extent of lesions and the potential for further spread. The patient experienced mild, transient chest pains the morning after hospitalization. To assess the possibility of postinfection myocarditis (3), an electrocardiogram was performed and the cardiac troponin level was measured. Both tests were normal, and the patient was discharged from the hospital on June 27. By July 6, when the patient had a follow-up examination, his lesions had healed without complication.

## Tertiary Vaccinia Case

The patient with the secondary vaccinia virus infection reported having experienced the perianal rash at the time he had sexual intercourse with a different male partner on June 22. The second male partner reported experiencing lesions on June 24. When interviewed, he reported no other recent sexual partners and said he had never been vaccinated against smallpox.

The second male partner sought care on June 25 from the same infectious disease specialist who had evaluated the secondary patient. He reported experiencing malaise, sore throat, and nasal congestion the day after sexual contact with the secondary patient. On physical examination, eight raised papular lesions were noted on his penis, and one was observed on the right forearm, all suspicious for vaccinia virus infection. Swab specimens of the lesions tested positive by PCR for nonvariola *Orthopoxvirus* by the County of San Diego Public Health Laboratory.

On initial evaluation and interview, the patient with tertiary infection reported no history of skin disease; however, three days later he recalled having had eczema as a child. By this time, his lesions had become more numerous and progressively painful. Re-examination on June 28 revealed 11 umbilicated lesions: eight previously noted on the penis, one previously noted on the arm, and two new lesions on the groin. In light of the number, location, and extent of lesions and the recently recalled history of eczema, CDC released VIGIV for the patient with tertiary infection, and he was admitted to the hospital. VIGIV was administered in the hospital on June 29 with no adverse effects. Two days later, five additional lesions developed on the penis and scrotum. No further lesions developed after July 1, and the patient was discharged from the hospital on July 2. At a follow-up examination on July 11, the lesions were found to have healed without further complication.

## Confirmation of Antivaccinia Antibodies

A serum specimen was drawn from each of the two patients before and 48 hours after VIGIV administration to ensure detectable levels of immune globulin. The secondary patient had detectable antivaccinia antibodies in the pre-VIGIV serum specimen, and the tertiary patient did not. Both patients had detectable antivaccinia antibodies in post-VIGIV serum specimens.

## Smallpox Vaccinee

The vaccinee was identified as a civilian who had received his first smallpox vaccine in June 2012 under the Department of Defense smallpox vaccination program. At a routine follow-up examination to check the inoculation site on June 13, the vaccinee reported not having kept the site covered as instructed. Clinic staff members again instructed him to keep the lesion covered and repeated the instructions provided previously to reduce the risk for vaccinia transmission to others. The vaccinee experienced the expected pustular lesion at the inoculation site on his left upper arm and did not experience any secondary lesions or complications. The vaccinee was interviewed on July 9, during epidemiologic investigation of the secondary and tertiary patients. He confirmed that no secondary lesions had occurred and reported that the secondary patient was his only sexual contact during the infectious window, days 2–30 after receiving the smallpox vaccine.

## Prevention Recommendations

Interviewed at the time of illness, neither patient reported having additional sexual contacts or living with persons who might be at risk for complications from vaccinia infection. Persons at risk include those who are immunosuppressed, pregnant women, or persons with a history of atopic dermatitis (2). The patient with tertiary infection did not go to work on the day he experienced symptoms and returned when his lesions were healed adequately. Both patients wore contact lenses, which can pose a hazard for ocular autoinoculation with the virus. Neither patient wore eyeglasses in lieu of contact lenses. Extensive patient instructions were provided to prevent autoinoculation and further transmission to contacts. Recommendations focused on refraining from sexual or other intimate contact until lesions had healed completely, the importance of hand hygiene (especially when handling contact lenses), managing infectious fomites (e.g., clothing, bedding, and towels), and lesion care. The military clinic that administered the smallpox vaccine was contacted to ensure vaccinees were provided the required instructions regarding preventing virus transmission to others (1). No further transmission of vaccinia virus by the smallpox vaccinee or the secondary or tertiary patient has been reported.

What is already known on this topic?

Unintended transmission of vaccinia virus can occur through contact with civilian and military personnel vaccinated under the U.S. Department of Defense smallpox vaccination program.

What is added by this report?

Sexual contact with a civilian recently vaccinated against smallpox resulted in secondary and tertiary transmission of vaccinia virus. Virus transmission resulted in illness, multiple lesions in the genital and perianal areas, and singular lesions in other sites. Vaccinia immune globulin intravenous (VIGIV) was administered to both the secondary and tertiary patient to prevent worsening and spread of lesions. Both patients recovered.

What are the implications for public health practice?

This report highlights the potential for further transmission of vaccinia virus beyond direct sexual contacts of smallpox vaccinees and the importance of vaccinee compliance with covering the inoculation site. VIGIV might be indicated in patients with vaccinia lesions in genital areas to prevent further lesion spread.

### Editorial Note

In 2002, the U.S. Department of Defense resumed smallpox vaccination for designated military personnel, civilian employees, and contractors. The smallpox vaccine licensed for use in the United States contains live vaccinia virus. The CDC Laboratory Response Network supports a nonvariola *Orthopoxvirus* test that can identify vaccinia and other nonvariola orthopoxviruses in clinical specimens. Since the Department of Defense resumed smallpox vaccination, cases of secondary transmission of vaccinia virus from military smallpox vaccinees have been reported among intimate (4–6), sports-related (7), and household contacts (4). Tertiary transmission has been reported among household and sports contacts (4,8) and from mother to child through breastfeeding (9). This case report is the first reported instance of tertiary vaccinia transmission through sexual contact.

Contraindications for routine, nonemergency vaccination against smallpox include presence of atopic dermatitis or any history of atopic dermatitis, other exfoliative skin conditions, pregnancy, immunosuppression, or living in a household with a person who has any of these conditions (2). Eczema vaccinatum is an immune-mediated adverse reaction to vaccinia virus that can occur in persons with ongoing or past history of atopic dermatitis (10). Because the term eczema often is applied to different dermatologic diseases, assessing whether a patient has a history of atopic dermatitis versus another eczematous skin condition can be difficult (10).

VIGIV is the only licensed treatment available for complications from vaccinia virus infection. Indications for its use include treatment or mitigation of aberrant vaccinia infections that pose a particular hazard (e.g., inadvertent inoculation of the eyes or mouth) as well as eczema vaccinatum, progressive or severe generalized vaccinia infections, and other skin conditions.* The majority of adverse vaccinia reactions do not require treatment beyond supportive care. VIGIV is reserved for patients with serious clinical disease or for those at risk for experiencing severe disease. CDC is the sole source of VIGIV for civilians. All suspected cases of contact-transmitted vaccinia should be reported to state or local health departments and to the Vaccine Adverse Events Reporting System (http://vaers.hhs.gov).

The secondary and tertiary patients in this investigation experienced symptoms of systemic illness, localized proliferation of vaccinia lesions, and singular lesions at locations remote from the principal inoculation sites. Lesions in the genital and perianal areas are challenging for preventing autoinoculation and further local inoculation by clothing and other fomites. A possible history of atopic dermatitis was concerning; however, the major reason why the decision was made to administer VIGIV to both patients was because of lesion location, number, and progression.

Both patients sought medical care early in the course of disease, which also contributed to the decision to administer VIGIV. Early presentation provided an opportunity to supply antivaccinia antibodies when the patients’ immune systems were beginning to respond to the infection. These case reports describe secondary and tertiary transmission of vaccinia virus through sexual contact, highlighting the potential for vaccinia infections to spread beyond immediate intimate contacts of smallpox vaccinees. The illness experienced by the two patients and the potential for further contact transmission underscores the importance of smallpox vaccinee compliance with covering the inoculation site and instruction regarding the particular hazards of vaccinia transmission to genital and perianal areas.

## Footnotes

* Additional information available at http://www.fda.gov/biologicsbloodvaccines/bloodbloodproducts/approvedproducts/licensedproductsblas/fractionatedplasmaproducts/ucm179513.htm.
